# Cytochrome *c* Is Tyrosine 97 Phosphorylated by Neuroprotective Insulin Treatment

**DOI:** 10.1371/journal.pone.0078627

**Published:** 2013-11-05

**Authors:** Thomas H. Sanderson, Gargi Mahapatra, Petr Pecina, Qinqin Ji, Kebing Yu, Christopher Sinkler, Ashwathy Varughese, Rita Kumar, Melissa J. Bukowski, Renee N. Tousignant, Arthur R. Salomon, Icksoo Lee, Maik Hüttemann

**Affiliations:** 1 Department of Emergency Medicine, Wayne State University School of Medicine, Detroit, Michigan, United States of America; 2 Cardiovascular Research Institute, Wayne State University School of Medicine, Detroit, Michigan, United States of America; 3 Center for Molecular Medicine and Genetics, Wayne State University School of Medicine, Detroit, Michigan, United States of America; 4 Department of Biochemistry and Molecular Biology, Wayne State University School of Medicine, Detroit, Michigan, United States of America; 5 Institute of Physiology and Center for Applied Genomics, Academy of Sciences of the Czech Republic, Prague, Czech Republic; 6 Department of Molecular Biology, Cell Biology, and Biochemistry, Brown University, Providence, Rhode Island, United States of America; 7 College of Medicine, Dankook University, Cheonan-si, Chungcheongnam-do, Republic of Korea; Consiglio Nazionale delle Ricerche, Italy

## Abstract

Recent advancements in isolation techniques for cytochrome *c* (Cyt*c*) have allowed us to discover post-translational modifications of this protein. We previously identified two distinct tyrosine phosphorylated residues on Cyt*c* in mammalian liver and heart that alter its electron transfer kinetics and the ability to induce apoptosis. Here we investigated the phosphorylation status of Cyt*c* in ischemic brain and sought to determine if insulin-induced neuroprotection and inhibition of Cyt*c* release was associated with phosphorylation of Cyt*c*. Using an animal model of global brain ischemia, we found a ∼50% decrease in neuronal death in the CA1 hippocampal region with post-ischemic insulin administration. This insulin-mediated increase in neuronal survival was associated with inhibition of Cyt*c* release at 24 hours of reperfusion. To investigate possible changes in the phosphorylation state of Cyt*c* we first isolated the protein from ischemic pig brain and brain that was treated with insulin. Ischemic brains demonstrated no detectable tyrosine phosphorylation. In contrast Cyt*c* isolated from brains treated with insulin showed robust phosphorylation of Cyt*c*, and the phosphorylation site was unambiguously identified as Tyr97 by immobilized metal affinity chromatography/nano-liquid chromatography/electrospray ionization mass spectrometry. We next confirmed these results in rats by in vivo application of insulin in the absence or presence of global brain ischemia and determined that Cyt*c* Tyr97-phosphorylation is strongly induced under both conditions but cannot be detected in untreated controls. These data suggest a mechanism whereby Cyt*c* is targeted for phosphorylation by insulin signaling, which may prevent its release from the mitochondria and the induction of apoptosis.

## Introduction

Brain ischemia, caused by stroke or cardiac arrest, results in extensive brain damage and is a major cause of death and disability [1]. In an attempt to reduce this neurologic damage, many investigators have utilized therapies that stimulate cell survival signaling pathways [2], [3]. Our studies have identified insulin as a potent growth factor that induces cell survival signaling and prevents neuronal apoptosis following global brain ischemia [4], [5]. The mechanism of insulin-mediated neuroprotection is independent of its effect on lowering serum glucose [6] but relies on its ability to induce cell survival signals [4], [5]. We have previously shown that administration of insulin immediately following an ischemic insult activates the PI3K-Akt cell survival pathway, inhibits Bax translocation to the mitochondria, promotes favourable Bcl-2 family protein interactions on the mitochondria, inhibits Cyt*c* release, and importantly, protects hippocampal structure and function [4], [5]. However, while these studies revealed insulin induced cell signaling events associated with inhibition of Cyt*c* release and attenuation of cell death, the specific cell signaling events responsible for this effect remain to be elucidated.

Release of Cyt*c* from the mitochondria is a critical event in initiation of cell death following global brain ischemia [7]–[10]. Once released into the cytosol, Cyt*c* binds to Apaf-1 and procaspase-9 to form the apoptosome thereby initiating apoptotic cell death [11]. These events have been proven critical to the delayed neuronal death that occurs following an ischemic insult to the brain [10], [12]. Although there are many proposed mechanisms of Cyt*c* release, results are often contradictory in the setting of brain reperfusion and the precise mechanism remains unknown. Moreover, we must further our knowledge of cell survival signaling that prevent post-ischemic release of Cyt*c* to improve our development of therapies.

Traditional studies of isolated Cyt*c* did not preserve the *in vivo* phosphorylation state during protein purification. Recent studies by our group have demonstrated that mammalian Cyt*c* can be post-translationally modified by tyrosine phosphorylation at two distinct residues in a tissue-specific manner [13]–[15]. These results introduced a novel concept, that Cyt*c* is targeted by cell signaling by yet unidentified mitochondrial tyrosine kinases. Phosphorylation of Cyt*c* occurs at Tyr48 in liver and Tyr97 in heart tissue, which causes partial inhibition in the reaction with isolated cytochrome *c* oxidase [13], [14], or ‘controlled’ respiration that is conducive to healthy electron transfer rates. Two more phosphorylation sites were recently mapped on Cyt*c* from human skeletal muscle in a high throughput mass spectrometry study [16]. Those sites are Thr28 and Ser47, but their effect on Cyt*c* function has not been studied. Notably, in all the tissues investigated using conditions that preserve phosphorylation state, Cyt*c* has been shown to be phosphorylated. Based on these findings we proposed a new model in which Cyt*c* phosphorylation maintains controlled electron transfer rates, thereby preventing hyperpolarization of the mitochondrial membrane potential and limiting ROS production [17], [18]. In contrast, under stressed conditions Cyt*c* and other OxPhos components become dephosphorylated leading to maximal flux in the ETC resulting in the mitochondrial membrane potentials exceeding 140 mV, a condition known to promote excessive mitochondrial ROS generation [19].

The role of Cyt*c* in mitochondrial type II apoptosis may also be modulated by Cyt*c* phosphorylation. Interestingly, phosphomimetic substitution of Cyt*c* Tyr48 with Glu completely abolished the ability of this protein to induce apoptosis *in vitro*, suggesting that cell signaling can regulate the execution of apoptosis at the level of Cyt*c*. Additionally, this same alteration reduces the binding affinity of Cyt*c* for the inner mitochondrial membrane lipid, cardiolipin [15]. Dissociation of Cyt*c* from cardiolipin is a prerequisite for its release into the cytosol [20]–[22]. These effects of Cyt*c* phosphorylation on respiration and apoptosis, along with the anti-apoptotic effects of insulin administration, led us to investigate the role of Cyt*c* phosphorylation in the neuroprotection conferred by insulin following an ischemic insult to the brain.

## Materials and Methods

### Model of global brain ischemia

Chemicals were purchased from Sigma unless otherwise stated. Animal experiments in this study were approved by the Wayne State University Animal Investigation Care and Use Committee and conform to the guidelines on the ethical treatment of animals presented in the National Research Council's *Guide for the Care and Use of Laboratory Animals*, 8^th^ Edition and stated in the US Government Principles for the Utilization and Care of Vertebrate Animals Used in Testing, Research, and Training. Animal numbers were kept to a minimum consistent with statistical significance. Global brain ischemia of 8 minutes duration was induced using bilateral carotid occlusion and hypotension as described by Smith et al. [23] and modified by Sanderson et al. [4], [24]. Sprague Dawley rats weighing 325–375 grams were anesthetized with halothane, orotracheally intubated, and mechanically ventilated. Femoral artery and vein catheters were inserted and arterial blood pressure was continually monitored through the arterial line. A midline incision was made in the ventral neck, and the carotids were bluntly dissected. Ischemia was induced by rapidly withdrawing blood from the femoral artery to achieve a mean arterial pressure of 30±1 mmHg within 1 min. Both carotids were then occluded with microaneurysm clips. After 8 minutes, the clips were removed and the withdrawn blood, maintained at 37° C, was reinfused to achieve a mean arterial pressure of 70–90 mmHg within 2 min. This model consistently causes ∼90% loss of pyramidal neurons in the CA1 hippocampus. Immediately following ischemia, an intravenous bolus of either insulin (20 U/kg) or saline vehicle was infused. This dose of insulin was selected based on our previous studies that demonstrated it induced cell survival signaling in the brain [5], and is maximally neuroprotective [4] and these effects were not achieved with 2 or 10 U/kg insulin. Blood glucose was frequently tested and intravenous infusions of 50% dextrose were given as needed. This regimen prevented serum glucose from dropping below normal levels (105 mg/dL) during reperfusion, and was not needed after 2 h. Sham-operated control animals underwent the entire surgery excluding blood withdrawal and carotid occlusion and were kept under anesthesia for 2 h. For Western blot analysis, after 24 h of reperfusion animals were transcardially perfused with ice-cold isotonic saline, and the brain was rapidly removed. The hippocampal isolation began with a midline incision along the longitudinal fissure through the corpus callosum followed by careful separation of the telencephalon from the diencephalon. The hippocampus was then rapidly dissected and homogenized. When brains were used for immunofluorescence, the rats were transcardially perfused with 4% paraformaldehyde, postfixed for an additional 2 h, cryoprotected in 30% sucrose in PBS, and then frozen in isopentane and dry ice.

### Subcellular fractionation

The hippocampal tissue was weighed, homogenized using a Dounce homogenizer in 1∶5 (wt∶vol) isotonic HEPES isolation media (20 mM HEPES pH 7.4, 250 mM sucrose, 10 mM KCl, 1.5 mM MgCl_2_, 1 mM EGTA, 1 mM EDTA) supplemented with protease inhibitors (0.2 mM phenylmethylsulfonylfluoride (PMSF), 1 µg/mL pepstatin, 10 µg/mL aprotinin, 10 µg/mL leupeptin, 12.5 µg/mL calpain inhibitor I), and then centrifuged at 750 x *g* for 10 min. The resulting supernatant was centrifuged at 14,000 x *g* for 10 min and the pellet taken as the crude mitochondrial fraction. The remaining supernatant was centrifuged at 100,000 x *g* for 60 min and the supernatant was taken as the cytosolic fraction. Purity of the preparation was assessed by immunoblotting each fraction with antibodies against the mitochondrial marker CcOIV (cytochrome *c* oxidase subunit IV, Invitrogen, Carlsbad, CA) and the cytosolic marker β-actin (Cell Signaling, Danvers, MA), and all data were normalized to the appropriate marker.

### Immunofluorescence labeling

After 8 min of ischemia followed by 14 days of reperfusion, rat brains were fixed as described above, cryoprotected, frozen, and cryosectioned at 20 µm. Sections were subjected to triple-label immunofluorescence or Cresyl violet staining as described previously [4]. Briefly, the sections were quenched with 3% peroxide, blocked with 5% BSA, and incubated overnight with primary antibodies for Iba-1 (ab5076, Abcam, Cambridge, MA), then GFAP (ab16997, Abcam), and finally neuronal nuclei (NeuN, MAB377, Millipore, Billerica, MA). AlexaFluor conjugated secondary antibodies were used: Iba1- AlexaFluor 488 anti-goat, GFAP- AlexaFluor 647 anti-rabbit, NeuN-AlexaFluor 546 anti-mouse. Sections were treated with copper sulfate in ammonium acetate buffer to quench endogenous autofluorescence of the brain tissue. Immunofluoresence images were acquired on a Leica (Wetzlar, Germany) LSM510 confocal microscope, under a 63X oil-immersion objective. A series of 10 optical sections were taken every 0.25 µm in the z-plane, stacked into z-stacks of 2.5 µm, and shown as a z-projection of the total z-stack using ImageJ software (National Institutes of Health, Bethesda, MD).

### Isolation of pig and rat brain Cyt*c*

Pig brains were obtained as discarded tissue from a slaughterhouse (Wolverine Packing Company, Detroit) with consent for use and immediately frozen on dry ice. Pooled tissue (1.8 kg) was ground using a commercial meat grinder and split into two equal fractions. The first (control) fraction was supplemented with 3 L of extraction buffer (100 mM KPi; pH 4.5, adjusted with acetic acid) and immediately homogenized using a commercial blender to extract Cyt*c*. The acidic extraction and subsequent isolation of Cyt*c* was performed as described [14] with modification as detailed below. The other fraction was incubated with 1 L of buffer A (250 mM sucrose, 20 mM Tris (pH 7.4), 2 mM EGTA, 1 mM PMSF) prewarmed to 30 °C. The suspension was supplemented with 1 µM insulin and incubated for 25 min at 30°C under stirring to allow adequate tissue oxygenation and cell respiration. pH was measured every two min and readjusted if necessary. After insulin treatment the suspension was transferred to 2 L of ice-cold extraction buffer (100 mM KPi final concentration), homogenized as above, and pH was adjusted to 4.5. The control and insulin-treated suspensions were stirred for 12 h at 4°C to extract Cyt*c*, and Cyt*c* purifications were performed side-by-side. Each suspension was centrifuged (27,000 *x* g, 40 min), and the supernatants containing Cyt*c* were adjusted to pH 7.5 and at the same time supplemented with unspecific phosphatase inhibitors KF (10 mM) and activated vanadate (1 mM) to prevent dephosphorylation [13]. At pH 7.5 more proteins precipitated and another centrifugation was carried out as above. The Cyt*c*-containing supernatants were subjected to DE52 anion exchange column and CM52 cation exchange column chromatography (Whatman, Jersey City, NJ) as described [14]. To increase purity, the DE52 and CM52 ion exchange steps were repeated. Cyt*c* was concentrated under vacuum to 2 mL, desalted via Sephadex G50 gel filtration, and subjected to HPLC purification (Jasco Inc., Easton, MD) using a Macrosphere™ GPC column (7.5×300 mm; 100 Å, Grace, Deerfield, IL)[14]. Cyt *c* was concentrated, desalted, and stored at −80°C.

For small scale rat brain Cyt*c* isolation 8 brains (about 16 g total) were combined for each condition. To improve yield, the ratio of extraction buffer volume (800 mL) to tissue was increased compared to the large scale pig brain isolation procedure. The subsequent steps were performed as above except for the DE52 anion exchange column and CM52 cation exchange column chromatography steps, which were each performed only once.

### Western analysis

For Western blot of subcellular fractions, equal amounts of protein (5 µg for mitochondrial fractions or 20 µg for cytosolic) were separated using SDS-PAGE, and then transferred to nitrocellulose. Membranes were incubated in primary antibody (1∶500 to 1∶1,000 according to manufacturer's specifications) followed by secondary antibody (1∶10,000 dilution). Antibody binding was detected using the enhanced chemiluminescence technique (GE Healthcare, Piscataway, NJ). Primary antibodies used for immunoblotting were: Cyt*c* (BD Biosciences, San Diego, CA), β-actin (Cell Signaling, Danvers, MA), and CcOIV (Invitrogen, Carlsbad, CA).

For Western analysis of isolated Cyt*c*, SDS-PAGE was carried out using a 12% Tris-Tricine gel. After protein transfer to a PVDF membrane (Bio-Rad, Hercules, CA), Western analysis was carried out as described [14] with a 1∶5,000 dilution of anti-phospho tyrosine (4G10, Millipore, Bilerica, MA) followed by a 1∶10,000 dilution of anti-mouse IgG horseradish peroxidase-conjugated secondary antibody (GE Healthcare). EGF-stimulated A431 total cell lysate (Upstate, Bilerica, MA) was included as a positive control and ovalbumin as a negative control. Signals were detected as above. Anti-phosphoserine and anti-phosphothreonine antibodies were sets of four (1C8, 4A3, 4A9, and 16B4), and three (1E11, 4D11, and 14B3) individual monoclonal antibodies (EMD Biosciences, Gibbstown, NJ) and were used as described above.

### Mass spectrometry

Forty µg of purified and desalted Cyt*c* were reconstituted with 40 µL of 100 mM NH_4_HCO_3_ (pH 8.9) and then reduced with 10 mM DTT for 1 h at 56 °C, followed by alkylation with 55 mM indoacetamide for 1 h at room temperature in the dark. Proteins were digested with affinity purified and TPCK treated trypsin (Promega, Madison, WI) at a trypsin:protein ratio of 1∶100 (w/w) overnight at 37 °C. Digests were enriched for phosphopeptides using PhosTio Kit (GL Science, Tokyo, Japan). Briefly, PhosTio Tips were rinsed with Buffer A (8% TriFluoroacetic Acid, 90% Acetonitrile) and Buffer B (25% Lactic Acid, 6% TFA, 67.5% Acetonitrile). Then samples were diluted with 1.5-fold Buffer B and loaded onto the tips two times. Tips were rinsed with Buffer B once and Buffer A three times. Peptides were eluted from the TiO_2_ resin using 50 µL of 1% NH_4_OH in water and 50 µL of 1% NH_4_OH in 40% acetonitrile sequentially. Eluted peptides were further acidified by adding 4 µL of 50% acetic acid and dried to completeness.

The enriched phosphopeptide samples were reconstituted in 10 µL of 0.1 M acetic acid in water and loaded on a 75 µm I.D. precolumn packed with 3 cm of 5 µm Monitor C18 particles and eluted with a reversed-phase gradient (0–70% acetonitrile in 30 min) into the mass spectrometer (Linear Trap Quadrupole-Fourier Transform (LTQ-FT), Thermo Fisher Scientific, Waltham, MA) through an analytical column (360 µm outer diameter ×75 µm inner diameter-fused silica capillary with 12 cm of 5 µm Monitor C18 particles with an integrated 4 µm-ESI emitter tip fritted with polymer). Static peak parking was performed via flow rate reduction from 200 nL/min to 40 nL/min when peptides began to elute as judged from a bovine serum albumin peptide scouting run. Using a split flow configuration, an electrospray voltage of 2.0 kV was applied as described [25]. Spectra were collected in positive ion mode and in cycles of one full MS scan in the Fourier Transform (m/z 400–1800) followed by data-dependent MS/MS scans in the LTQ (0.2 s each), sequentially of the nine most abundant ions in each MS scan with charge state screening for +1, +2, +3 ions and dynamic exclusion time of 30 s. The automatic gain control was 1,000,000 for the FTMS scan and 10,000 for the ion trap mass spectrometry scans. The maximum ion time was 100 ms for the ion trap mass spectrometry scan and 500 ms for the FTMS full scan. FTMS resolution was set at 100,000.

MS/MS spectra were assigned to peptide sequences from the NCBI non-redundant protein database sliced in Bioworks 3.1 for porcine proteins and searched with the SEQUEST algorithm. SEQUEST search parameters designated variable modifications of +79.9663 Da on Ser, Thr, and Tyr (phosphorylation). Identified phosphopeptide spectra of interest were manually verified.

## Results

### Insulin inhibits cytochrome *c* release and protects the CA1 hippocampus following global brain ischemia

We have previously shown that Cyt*c* is tyrosine-phosphorylated *in vivo* in heart and liver tissue, and that these phosphorylations lead to healthy, controlled (i.e., lower) cell respiration rates, which may prevent the execution of the apoptotic pathway [13]–[15]. Since the molecular mechanism of insulin's neuroprotective effect remained unclear we hypothesized that insulin treatment leads to phosphorylation of Cyt*c*.

**Figure 1:**
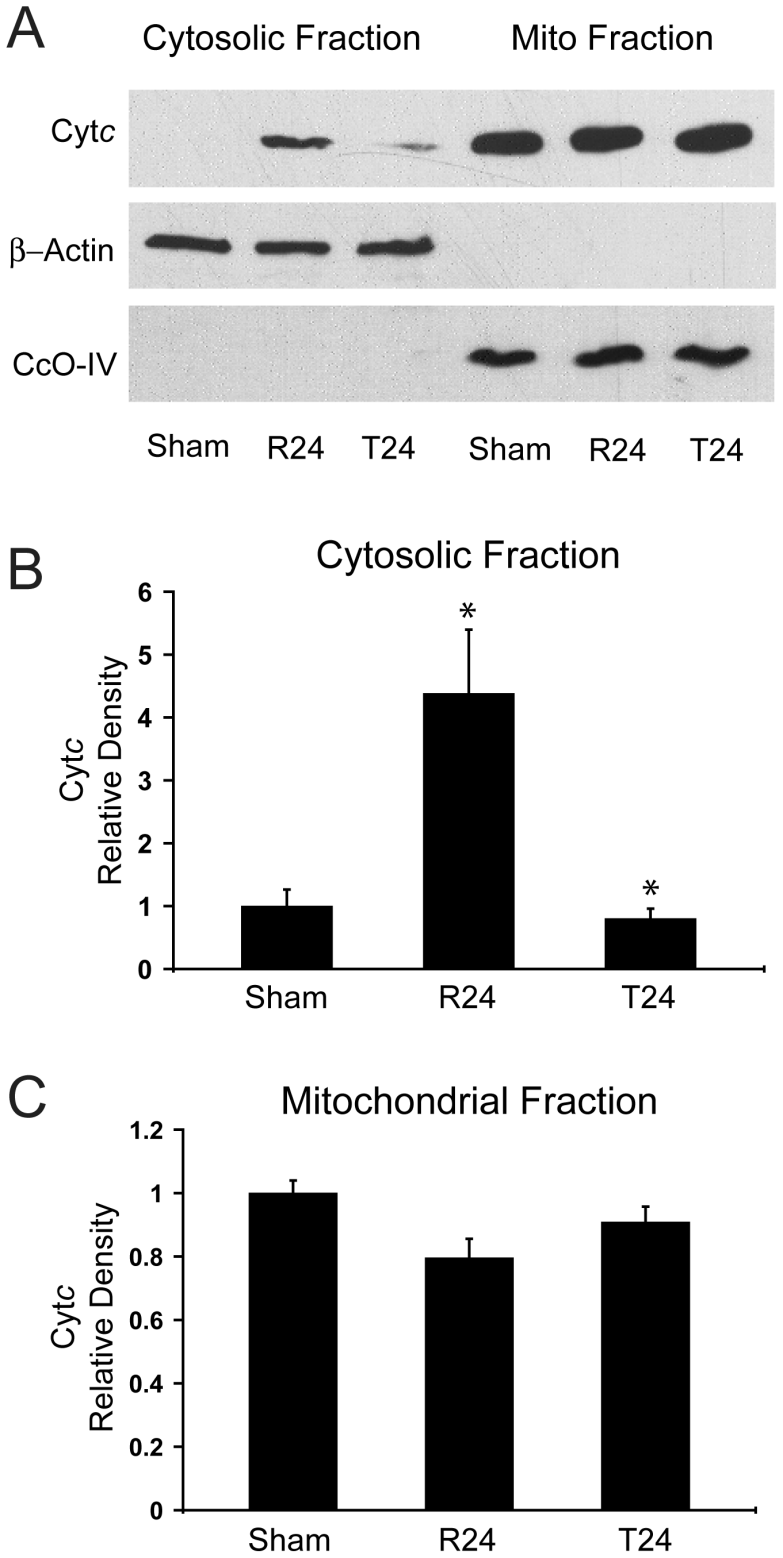
Cytochrome *c* release is inhibited by insulin administration. (A) Sham operated control animals (Sham, n = 3) show faint detectable amounts of Cyt*c* in the cytosolic fraction of hippocampal CA1 neurons. After 8 min of ischemia followed by 24 h of reperfusion (R24, n = 5) cytosolic Cyt*c* increases ∼4 fold (p < 0.05) and there is a trend toward mitochondrial Cyt*c* decrease (p = 0.08), both indicative of mitochondrial Cyt*c* release. Animals exposed to 8 min of ischemia followed by 24 h of reperfusion with a single bolus of IV insulin at the onset of reperfusion (T24, n = 5) demonstrate a reduction of cytosolic Cyt*c* (p < 0.05) and increased mitochondrial Cyt*c* compared to the R24 controls. (B and C) Densitometric analyses of Western blot data (Mean +/−SEM, *p < 0.05).

**Figure 2:**
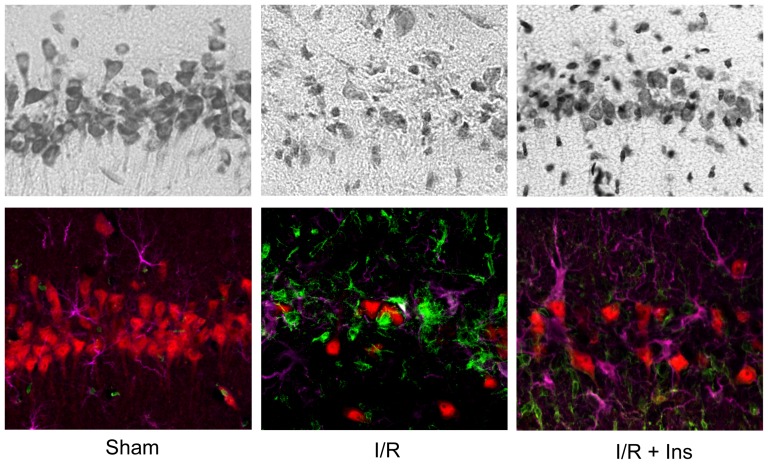
Insulin prevents neuronal cell death in the CA1 hippocampus following brain ischemia. Cresyl violet stained sections (top row) show CA1 hippocampus densely populated with pyramidal neurons in sham-operated controls (Sham), and triple-label immunofluorescence (bottom row) shows these cells to be NeuN-positive (red). After 8 min of global brain ischemia followed by 14 days of reperfusion (I/R, n = 5) there is a 90% loss of CA1 neurons and an increase in Iba-1-positive microglia and GFAP-positive astrocytes (green and magenta, respectively). Animals exposed to 8 min of ischemia followed by 14 days of reperfusion with a single bolus of insulin given at the onset of reperfusion (I/R + Ins, n = 4) demonstrate a 49% increase in NeuN-positive neurons (p < 0.05) while Iba-1 and GFAP-positive cells remain unchanged.

We first investigated the effect of insulin treatment on release of Cyt*c* from the mitochondria into the cytosol and subsequent cell death in a rat model of global brain ischemia/reperfusion injury. Cyt*c* release was detected by immunoblotting rat hippocampal homogenates fractionated into cytosolic and mitochondrial fractions with an anti-Cyt*c* antibody. Antibodies against cytochrome *c* oxidase subunit IV (CcOIV) and β-actin were used as loading controls and to determine the purity of mitochondrial and cytosolic fractions. We previously demonstrated that Cyt*c* release following global brain ischemia reaches a maximum level by 24 hours of reperfusion [4]. Therefore, we investigated whether insulin could prevent the peak Cyt*c* release at this late reperfusion interval, i.e., 24 hours of reperfusion, in order to determine if Cyt*c* release can be prevented and not just delayed. Western blot of cytosolic fractions showed a significant 4-fold increase in cytosolic Cyt*c* in the untreated controls (Fig. 1). This was associated with a trend towards a reduction in the overall mitochondrial pool of Cyt*c*, although this effect failed to reach statistical significance (p = 0.08). A single bolus of 20 U/Kg insulin administered at the onset of reperfusion prevented increased cytosolic Cyt*c* levels, maintaining cytosolic Cyt*c* levels similar to sham-operated controls (p = 0.98, Fig. 1; Sham vs. T24). To confirm if this effect on Cyt*c* release was associated with neuroprotection we used cresyl violet and triple-label immunofluorescence against a neuron marker (NeuN-Red), an astrocyte marker (GFAP-Magenta), and a microglial marker (Iba-1-Green). Sham-operated control animals exhibit a CA1 hippocampus densely populated with pyramidal neurons positive for NeuN, with few astrocytes and microglia (Fig. 2). Eight-minutes of ischemia followed by 14 days of reperfusion results in extensive loss of CA1 pyramidal neurons and an increase in GFAP-positive astrocytes and microglia/macrophages positive for Iba-1. The neuronal loss is partially prevented (49%) by insulin administration; however, gliosis remains unchanged.

### Insulin treatment leads to Cyt*c* tyrosine phosphorylation in the brain

**Figure 3:**
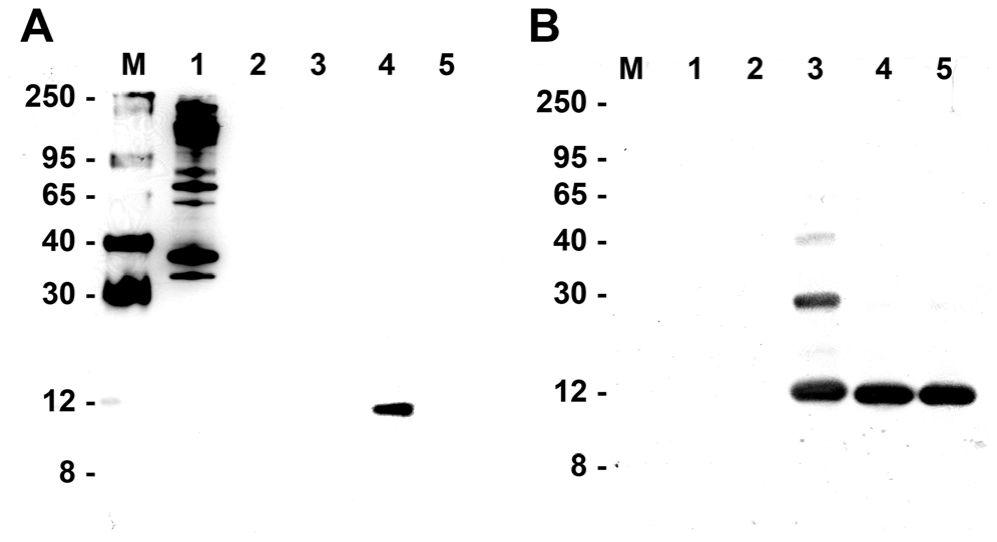
Insulin treatment leads to phosphorylation of brain cytochrome *c*. Ischemic pig brain tissue was treated +/− insulin following Cyt*c* purification under conditions that preserve protein phosphorylation. (A) Western analysis with an anti-phosphotyrosine antibody (4G10) indicates tyrosine phosphorylation of Cyt*c* after insulin treatment (lane 4), whereas Cyt*c* isolated without insulin treatment (lane 5) or obtained from a commercial source (lane 3, Sigma Cyt*c*) does not produce any signal. Lane M, protein size marker (kDa); lane 1, EGF stimulated A431 cell lysate (positive control for Western analysis); lane 2, ovalbumin (negative control for Western analysis). Western analysis with anti-phosphoserine and anti-phosphothreonine antibodies did not show any signal (not shown). (B) Control Western blot with an anti-Cyt*c* antibody shows similar loading. Samples in lanes 1–5 as denoted in A. Note that additional bands in Sigma Cyt*c* correspond to the Cyt*c* dimer and trimer that are sometimes observed depending on the batch.

To study phosphorylation of Cyt*c* the protein has to be isolated. Larger tissue amounts are necessary for this procedure and we therefore used pig brains as starting material. The brains were processed 30 min after the animals were killed, and Cytc was directly isolated to determine the ischemic phosphorylation state of Cyt*c*. To simulate neuroprotective insulin treatment, pig brains were incubated with insulin at a concentration of 1 µM for 25 min at 30°C (see Methods for details). Cyt*c* was then purified to homogeneity using our protocol that preserves phosphorylation. We performed Western analysis with anti-phospho-Ser/Thr/Tyr-specific antibodies and obtained a strong and specific signal with the anti-phospho-Tyr antibody (Fig. 3A, lane 4), whereas no signal was observed with the anti-phospho-Ser/Thr antibodies (not shown). Cyt*c* that was obtained from ischemic brain without insulin treatment did not show any phosphorylation with anti-phospho-Ser/Thr/Tyr antibodies, similar to commercially available Cyt*c* (lanes 5 and 3, respectively; similar amounts of protein were loaded as shown with an anti-Cyt*c* antibody (Fig. 3B)).

### Tyrosine 97 is phosphorylated after insulin treatment

**Figure 4:**
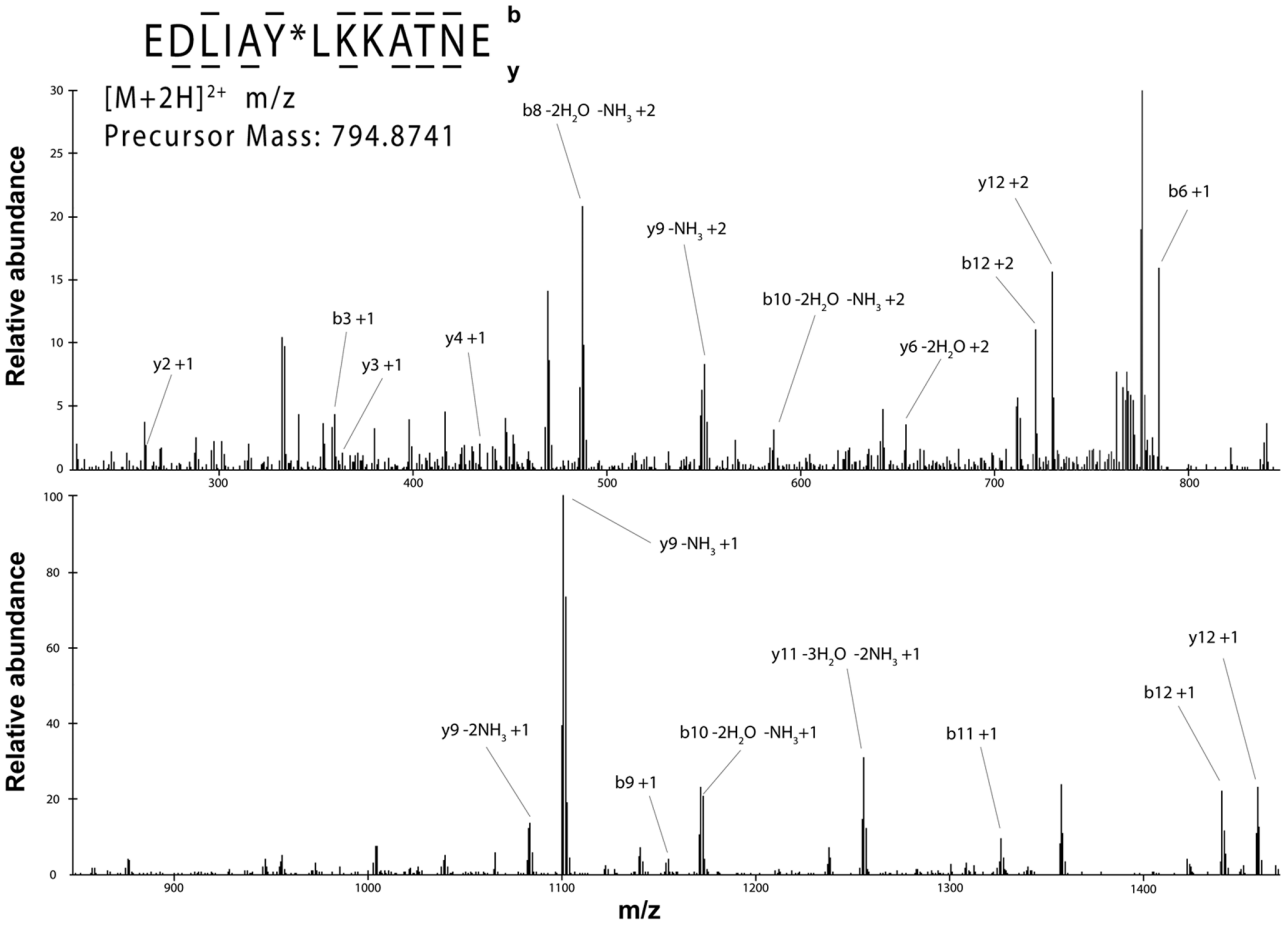
Nano-LC/ESI-MS/MS spectrum of EDLIApYLKKATNE. Peptides were eluted into the mass spectrometer by applying a HPLC gradient of 0–70% 0.1 M acetic acid/acetonitrile in 30 minutes. The mass spectrometer acquired top 9 data dependent ESI MS/MS spectra. The phosphorylation site was unequivocally assigned by fragment ions b6, b8, b9, b10, and y3, y4, y6. The sequence of the peptide was definitively assigned by b3, b6, b8, b9, b10, b11, b12, and y2, y3, y4, y6, y9, y10, y11.

To provide further proof that Cyt*c* was tyrosine phosphorylated after insulin treatment we determined the specific phosphorylation site. Isolated pig brain Cyt*c* was digested with trypsin and analyzed by immobilized metal affinity chromatography/nano-liquid chromatography/electrospray ionization mass spectrometry (IMAC/nano-LC/ESI-MS/MS). This method first enriched phosphorylated peptides via a TiO_2_ resin, and the eluted peptides were then subjected to tandem-MS for fragment examination. This analysis unambiguously revealed that Tyr97 was phosphorylated on Cyt*c* following insulin treatment on the peptide EDLIApYLKKATNE by fragment ions b6, b8, b9, b10, and y3, y4, y6 (Fig. 4). Importantly, no phosphorylation site was identified using control Cyt*c* that was directly isolated from ischemic brain tissue without insulin treatment, supporting findings by Western analysis (Fig. 3).

### Tyrosine 97 is phosphorylated after insulin treatment in vivo

**Figure 5:**
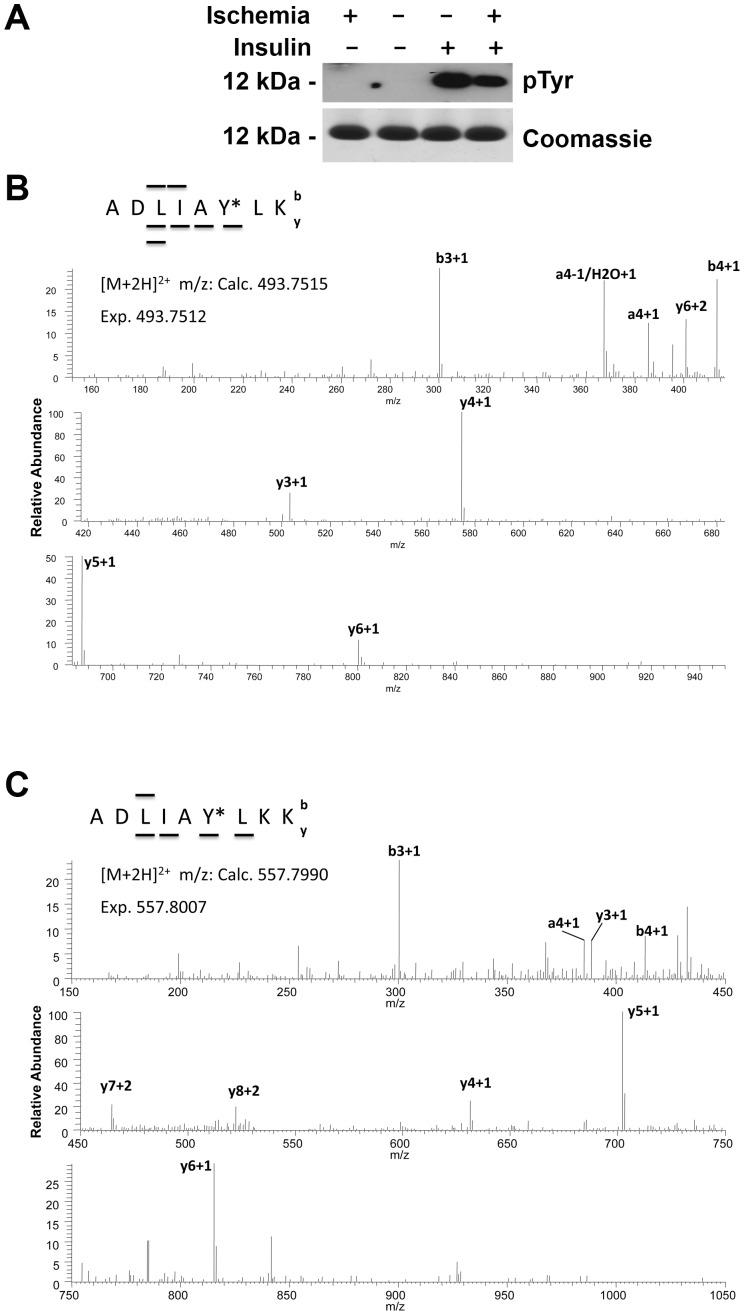
In vivo induction of cytochrome *c* Tyr97 phosphorylation in rat brain by insulin treatment. (A) Cyt*c* was isolated from rat brain after global brain ischemia (lane 1), from untreated control rats (lane 2), from sham-operated animals after insulin treatment (lane 3), and from rat brain after ischemia with insulin treatment (lane 4). Top, Western blot with an anti-phosphotyrosine antibody (4G10) reveals no detectable tyrosine phosphorylation of brain Cyt*c* under control conditions and after global brain ischemia (lanes 2 and 1, respectively), whereas tyrosine phosphorylation of Cyt*c* is strongly induced after insulin treatment (lane 3), but slightly reduced by ischemic stress (lane 4). Bottom, Coomassie gel shows equal loading (1 µg per lane) and purity of the isolated Cyt*c* species. (B) Nano-LC/ESI-MS/MS analysis of rat brain Cyt*c* after insulin treatment (corresponding to lane 3 in Fig. 5A) unambiguously identifies Tyr97 phosphorylation by fragment ions y3, y4, y5, and y6. The sequence of the peptide was definitively assigned by b3, b4, y2, y3, y4, y5, and y6. (C) Nano-LC/ESI-MS/MS analysis of rat brain Cyt*c* after global brain ischemia and insulin treatment (corresponding to lane 4 in Fig. 5A) unambiguously identifies Tyr97 phosphorylation by fragment ions y3, y4, y6, and y7. The sequence of the peptide was definitively assigned by b3, b4, y3, y4, y6, and y7.

In the previous section we showed that insulin applied to ischemic pig brain tissue in vitro leads to Tyr97 phosphorylation. To be fully consistent with the data of the release of Cyt*c* from the mitochondria, which is suppressed by insulin treatment (Fig. 1), we purified Cyt*c* from rat brains after the animals were subjected to the same treatment regimen. To do so we modified our standard purification protocol used for large tissue samples by increasing the volume of extraction buffer and by bypassing the second round of anion and cation exchange chromatography to further increase yield (see materials and methods section). The resultant Cyt*c* was of high purity (Fig. 5A, bottom). Consistent with the in vitro data (Figs. 3 and 4), rat brain Cyt*c* is not tyrosine phosphorylated under control conditions or after global brain ischemia (Fig. 5A, lanes 1 and 2). Importantly, in vivo insulin treatment leads to strong induction of tyrosine phosphorylation (Fig. 5A, lane 3), and this effect is persistent after global brain ischemia (Fig. 5A, lane 4). Analysis by mass spectrometry of both phosphorylated species after insulin treatment +/− global insulin treatment unambiguously revealed that the same residue, Tyr97, was phosphorylated in an insulin dependent manner (Fig. 5B and C). These in vivo data are consistent with the in vitro pig brain results showing phosphorylation of the same residue after insulin treatment (Fig. 4).

## Discussion

Studies investigating the role of Cyt*c* release following global brain ischemia have identified a critical role for Cyt*c* in neuronal cell death. Sugawara *et al.* demonstrated that release of Cyt*c* into the cytosol is associated with selectively vulnerable neuron populations in the brain [7]. Release of Cyt*c* into the cytosol is required for apoptotic cell death in human cells [26] and the intrinsic pathway of apoptosis is the primary route of cell death following global brain ischemia and reperfusion [27]. Many examples of Cyt*c* release during brain reperfusion exist and intervention at this point of cellular dysfunction is a potent method of neuroprotection [28], [29].

Insulin was first identified as a post-ischemic neuroprotective compound in the late 1980's [30], [31]. Its neuron-sparing effect was initially attributed to the ability of insulin to reduce post-ischemic hyperglycemia, a condition known to be neurodegenerative [30]. This was an intuitively appealing hypothesis given the enhanced damage caused by ischemia in patients with uncontrolled diabetes who lack insulin and are thereby hyperglycemic [32]. However, subsequent studies determined the neuroprotective effect of insulin to be independent of insulin's ability to reduce serum glucose levels [6]. Studies by our group have identified insulin's neuroprotective effect to be associated with stimulation of cell survival signaling cascades, most notably via PI3K, Akt, and inhibition of apoptosis [4], [5]. While multiple anti-apoptotic effects were found, no specific alterations were uncovered that demonstrated a direct link from insulin signaling to inhibition of Cyt*c* release.

Our results are the first to show that Cyt*c* can be targeted for phosphorylation in the brain, and that this phosphorylation is stimulated by the insulin signaling pathway in vitro (Fig. 3) and in vivo (Fig. 5). In both cases Tyr97 was unambiguously identified as the amino acid that is targeted for phosphorylation (Figs. 4 and 5). It is interesting to note that although insulin causes robust Tyr97 phosphorylation after global brain ischemia, the effect is less (reduced by 39%) compared to insulin treatment alone. This finding is consistent with our model [17], [18] that proposes hyperactivation of mitochondrial function after conditions of stress, such as ischemia, by stress-induced dephosphorylations of OxPhos proteins including Cyt*c*. We also show that Cyt*c* from a commercial source, likely isolated from ischemic cow heart tissue, is fully dephosphorylated. Cyt*c* in the dephosphorylated state promotes high (‘stressed’) electron transfer rates compared to phosphorylated Cyt*c*. Specifically, for Tyr97-phosphorylated Cyt*c* we have shown that the K_m_ of cytochrome *c* oxidase for Cyt*c* is 5.5 µM with sigmoidal kinetics, whereas dephosphorylation leads to a shift of the K_m_ to 2.5 µM and hyperbolic kinetics [13]. Cyt*c* isolated from heart showed a single phosphorylation site at Tyr97, similar to our results in brain reported here after insulin treatment. Both heart and brain are highly aerobic tissues that fully depend on energy provided by OxPhos.

Future work is necessary to reveal if Tyr97 phosphorylation also abolishes the ability of Cyt*c* to participate in apoptosis as was suggested for Tyr48-phosphorylated Cyt*c* based on studies with the phosphomimetic mutant Tyr48Glu Cyt*c* [15]. In the latter study, Tyr48Glu Cyt*c* mimicking Tyr48 phosphorylation was incapable of triggering downstream caspase activation, suggesting a safeguard mechanism to regulate apoptosis via cell signaling pathways. Therefore, insulin-triggered phosphorylation of Cyt*c* may confer neuroprotection in two ways: 1) a beneficial reduction of ETC flux, preventing a hyperpolarization of the mitochondrial membrane potential and thus ROS production, and 2) a change in its structure that interferes with apoptosome formation and/or activation. Indeed, preventing the interaction of Apaf-1 with Cyt*c* has been shown to be a potent neuroprotective strategy following brain ischemia [9]. Another study investigating the effect of Cyt*c* tyrosine nitration, which introduces a negative charge to the tyrosine and can thus be considered a phosphomimetic modification, demonstrated that Tyr97 nitration both reduces the binding affinity of Cyt*c* to Apaf-1 and inhibits the activation of downstream caspases [33].

In conclusion, the discovery that cell signaling controls Cyt*c* via phosphorylation expands the role of Cyt*c* from that of a passive electron carrier and apoptotic co-factor to an active player controlling cell fate decisions. Traditional studies attempting to prevent Cyt*c* release by preventing outer mitochondrial membrane permeabilization have yet to yield an effective neuroprotective agent. Specifically targeting Cyt*c* phosphorylation may provide a potent method for preventing cell death after ischemia/reperfusion injury in brain. Further studies are necessary to identify the kinase(s) and phosphatase(s) that act on Cyt*c*. These enzymes could then be specifically targeted to maintain Cyt*c* in the phosphorylated state during conditions of stress that would normally result in cell death.
